# Thymic Function and T-Cell Receptor Repertoire Diversity: Implications for Patient Response to Checkpoint Blockade Immunotherapy

**DOI:** 10.3389/fimmu.2021.752042

**Published:** 2021-11-24

**Authors:** Antonella Cardinale, Carmen Dolores De Luca, Franco Locatelli, Enrico Velardi

**Affiliations:** ^1^ Department of Pediatric Hematology and Oncology, Bambino Gesù Children’s Hospital, Istituto di Ricovero e Cura a Carattere Scientifico (IRCCS), Rome, Italy; ^2^ Department of Maternal and Child Health, Sapienza University of Rome, Rome, Italy

**Keywords:** immune reconstitution, thymus, immunotherapy, TCR repertoire diversity, T cells

## Abstract

The capacity of T cells to recognize and mount an immune response against tumor antigens depends on the large diversity of the T-cell receptor (TCR) repertoire generated in the thymus during the process of T-cell development. However, this process is dramatically impaired by immunological insults, such as that caused by cytoreductive cancer therapies and infections, and by the physiological decline of thymic function with age. Defective thymic function and a skewed TCR repertoire can have significant clinical consequences. The presence of an adequate pool of T cells capable of recognizing specific tumor antigens is a prerequisite for the success of cancer immunotherapy using checkpoint blockade therapy. However, while this approach has improved the chances of survival of patients with different types of cancer, a large proportion of them do not respond. The limited response rate to checkpoint blockade therapy may be linked to a suboptimal TCR repertoire in cancer patients prior to therapy. Here, we focus on the role of the thymus in shaping the T-cell pool in health and disease, discuss how the TCR repertoire influences patients’ response to checkpoint blockade therapy and highlight approaches able to manipulate thymic function to enhance anti-tumor immunity.

## Introduction

**Figure 1:**
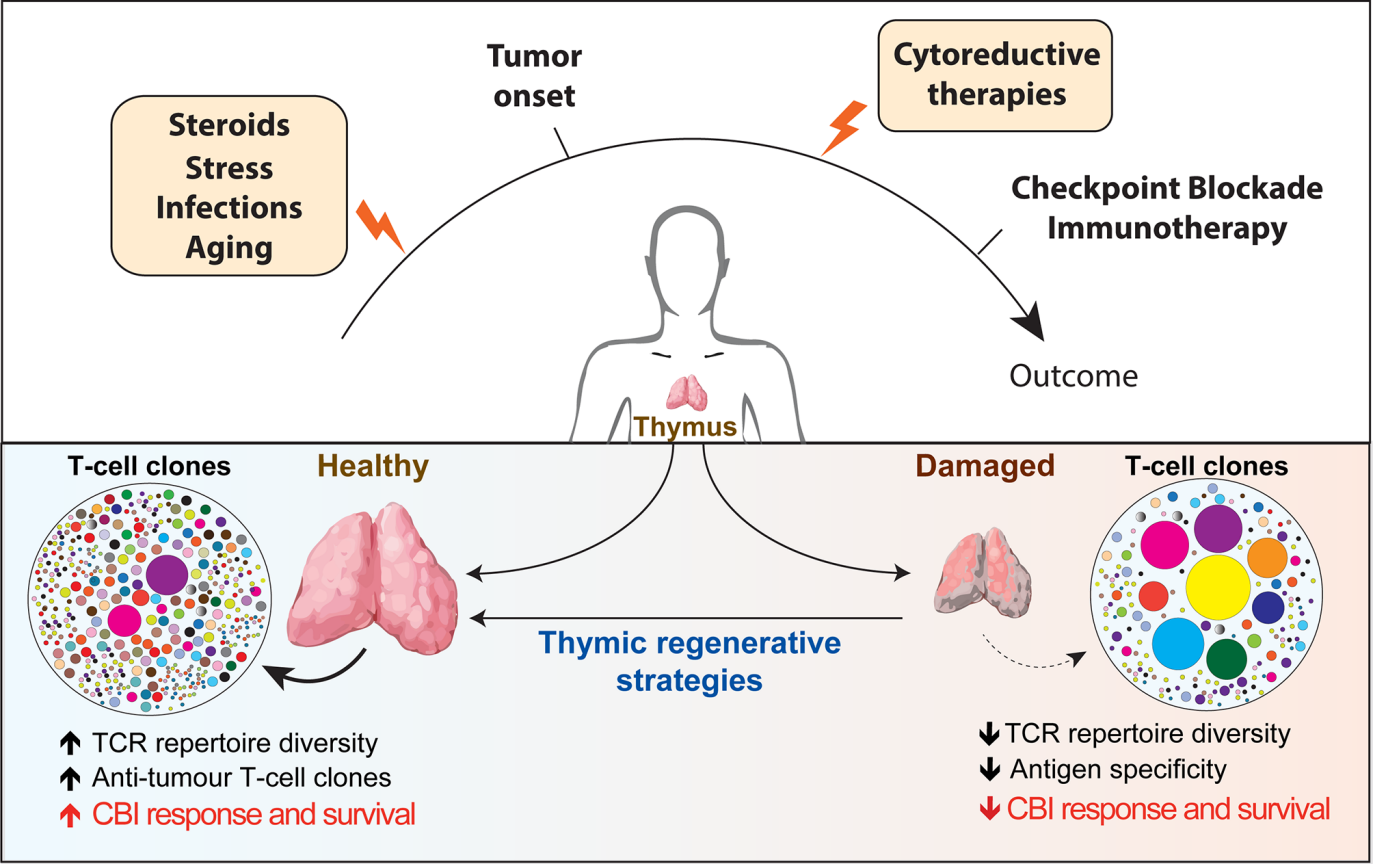
Overview of the factors affecting thymic function and their potential role in regulating patients’ response to checkpoint blockade immunotherapy. Thymus is particularly sensitive to negative insults that can come from infections, stress, cytoreductive therapies and the physiological process of aging (yellow boxes). The reduction in thymic functionality and in the TCR diversity impaired immune surveillance and may provide a supportive environment for tumors to elude T-cell-mediated response. Instead, a broader TCR repertoire in patients receiving CBI would increase the chance of tumor antigen recognition and favorable long-term clinical outcome. The use of regenerative factors aimed to boost thymic function could improve TCR repertoire diversity and have the potential to significantly extend the clinical efficacy of CBI. TCR, T cell repertoire; CBI, checkpoint blockade immunotherapy; SSA, sex steroids ablation.

Optimal immunological response to a large array of unknown antigens requires the presence of a diverse T-cell receptors (TCRs) repertoire, which represents the primary determinant for the likelihood of recognizing specific antigens (1). The thymus is the primary lymphoid organ with the exclusive role for generating and maintaining in the periphery a broadly diverse pool of T cells able to recognize tumor and pathogenic antigens. Once considered to take only a marginal part in maintaining a healthy immune system in adult life, the adult thymus plays a crucial role in sustaining the peripheral TCR repertoire diversity under physiological and clinical conditions. Thymic function and T-cell output are dynamic processes that can be severely compromised by acute immunological insults (resulting from infections, stress or antineoplastic therapies) and by chronic dysfunctions (such as the ones correlated to age-associated involution and recurrent infections). Suboptimal thymic function and skewed TCR repertoire can have profound immunological and clinical consequences for patients’ response to different forms of immunotherapy (**Figure 1**).

## Thymic Function and the Generation of a Diverse TCR Repertoire

During the process of T-cell development, thymocytes undergo a series of well-characterized and sequential developmental steps that ultimately lead to the formation of CD4 or CD8 single-positive T cells. These developmental steps are orchestrated by the crosstalk between bone marrow (BM)-derived T-cell progenitors and the supportive thymic stromal microenvironment, which primarily consists of thymic epithelial cells (TECs), endothelial cells (ECs), mesenchymal cells, dendritic cells and macrophages (2). A crucial step in T-cell development process is the generation of TCR molecules able to recognize antigenic peptides presented on heterologous cells. The recognition of a specific antigen is granted by three complementarity-determining regions (CDRs) of the TCR. The CDR3 regions are generated by somatic rearrangement between noncontiguous variable (V) and joining (J) gene segments for α and γ loci and between V, diversity (D), and J segments for the β and δ loci. The existence of multiple V, D and J gene segments in germline DNA allows the generation of a large variety of distinct CDR3 sequences that can be encoded (3). TCR rearrangement occurs in the thymic cortical and medullary regions where, respectively, the positive and negative selection of developing thymocytes occurs (4). Once the formation of a functional TCR is completed, T cells leave the thymus and enter the circulation where they impact the peripheral TCR diversity, specifically, of the *naïve* T-cell compartment.

The integrity of thymic function is essential for the generation of T cells with a diverse TCR. However, the thymus is particularly susceptible to negative insults that can come from infections, stress, acute and chronic Graft-*versus*-Host disease, cytoreductive therapies such as chemo and radiotherapy (5). These effects lead to a qualitative and quantitative decline in T-cell output with consequent restricted TCR repertoire diversity and impaired immune responses. At a specific time of an individual’s life, the peripheral diversity of TCR repertoire reflects and is shaped by multiple intrinsic and extrinsic factors, including the residual thymic functionality, previous response to pathogens, previous diseases and therapies, and many others.

In addition, the physiological process of aging has important effects on thymic function and TCR diversity. While the adult thymus can still generate new T cells up to the seventh decade of life, this process is severely compromised (6–8). It is well recognized that the size of peripheral *naïve* T-cell pool and the functionality of the immune system progressively decline with age (9). Particularly, aging impairs the normal process of T-cell development at multiple levels, including reduced numbers of lymphoid progenitors generated in the BM, decreased clonal deletion during negative selection (which increases the risk of releasing autoreactive T cells in the periphery), altered thymic microenvironment, reduced output of new T cells (6, 10). As a result, it has been estimated that only ~30–40% of elderly people are capable of mounting sufficient immune responses to the influenza vaccine (11). In addition, studies in pre-clinical models linked the skewed TCR repertoire occurring during aging to infection susceptibility (12). Although in healthy individuals thymic involution is not associated with any clinical consequences, the age-associated decline of thymic function significantly impairs the endogenous process of thymic repair following cytoreductive therapies further delaying the immune reconstitution in cancer patients (6).

Overall, reduction in thymic functionality and in the peripheral T-cell diversity are important contributors of the decline in immune surveillance observed in the elderly and this may eventually provide a supportive environment for infections and tumors to elude T-cell-mediated response. Even though there is a temporal correlation, the connection between decreased thymic function and increased incidence of cancers during age is still largely debated (13, 14).

## Impact of Thymic Function and TCR Diversity in Clinical Conditions

In several clinical conditions, damage to thymic function and changes in TCR repertoire diversity correlate with patients’ response to therapy and clinical outcome. In this section, we will provide a brief overview of how thymic functionality correlates with TCR diversity in human diseases and how TCR repertoire has been used to monitor and predict patient response to therapies.

Infections lead to severe thymic dysfunction, including reduced thymic output, altered thymic architecture and skewed TCR repertoire (15). Given that the degree of TCR diversity correlates with the chance of recognizing pathogenic antigens, the skewed TCR repertoire would probably represent a major factor in the reduced immune response to infections observed in HIV seropositive patients (16).

In patients affected by symptomatic SARS-CoV2 infection, lymphopenia, particularly in the CD8+ T cell compartment, has been shown to predict poor prognosis and can represent an early indicator for admission to the intensive care unit (17, 18). While there are not yet data on potential detrimental effects of SARS-Cov2 infection on thymic function, a recent study showed that Thymosin-α1 administration, which boosts immunity through thymic dependent and independent effects, increased survival of Covid-19 patients (19). Few studies are investigating the dynamic of TCR repertoire modification during infection demonstrating trends towards reduced TCR diversity in patients with pneumonia compared to those with mild disease (20). A clinical trial is ongoing to better characterize B- and T-cell repertoire and immune response in patients with acute and resolved Covid-19 infection (NCT04362865).

In patients receiving hematopoietic cell transplantation (HCT), impaired thymic function and suboptimal reconstitution of T-cell compartment have deleterious consequences. Thymic function is highly sensitive to conditioning regimens associated with the transplant procedure and delayed or defective recovery of its function has been linked to adverse clinical outcomes (21–24). Although mature T cells transferred with the graft or T cell clones resistant to conditioning procedure can expand and contribute to the recovery of the absolute lymphocyte counts early after HCT, the resulting T-cell immunity has a limited efficacy due to the skewed TCR repertoire. Low levels of tumor antigen-specific clonally expanded T cells are associated with higher risk of disease relapse (25). Indeed, higher TCR diversity has been correlated with lower relapse rates, presumably due to a greater probability of having T cell clones endowed with Graft-*versus*-Leukemia capacity (26). Similarly, delayed T-cell recovery and restricted TCR diversity post HCT are associated with increased risks of infection and leukemia relapse (27).

T-cell immunity is critical to control cancer occurrence and relapse; a more diverse TCR repertoire increases the likelihood of tumor-antigen recognition and mounting an effective immune response. For instance, reduced TCR diversity, when compared to healthy individuals, has been demonstrated in lung cancer patients (28). In addition, the TCR repertoire was particularly restricted in those patients carrying a more severe disease, which would indicate a defective antitumor immunity (28). In patients affected by cervical cancers, TCR repertoire diversity was lower than in patients with cervical intraepithelial neoplasia and healthy women, with a gradual decrease in TCR repertoire diversity during carcinogenesis and progression of the disease (29). Likewise, a recent study found that TCR repertoire diversity in renal cell carcinoma patients could predict better prognosis and the diversity was significantly higher in early disease stages. Interestingly, cytoreductive nephrectomy could restore TCR diversity, reduce T-cell exhaustion and induce mobilization of *naïve* T cells (30).

### Checkpoint Blockade Immunotherapy

Immunotherapy with monoclonal antibody-based immune checkpoint blockade (CBI) enhances the function of anti-tumor T lymphocytes in cancer patients, by targeting co-inhibitory signaling pathways.

Cytotoxic T-Lymphocyte Antigen 4 (CTLA4) is an early negative regulator of T-cell activation. It binds to CD80/CD86 (which provides co-stimulatory signal through CD28) and inhibits the acquisition of T-cell effector function. CTLA4 inhibits the priming of naive CD4+ T cells and reduces the function of memory CD8+ T cells. CTLA4 is also expressed on CD4+ FOXP3+ regulatory T cells (Tregs), contributing to their immunosuppressive property (31). Anti-CTLA4 monoclonal antibodies constrain Tregs immune suppression in the tumor microenvironment and enhance CD4+ and CD8+ T cells primary and memory function (32). Anti-CTLA4 monoclonal antibodies are used in several clinical settings, including stage III/IV melanoma, renal cell carcinoma, non-small-cell lung carcinoma (NSCLC) and prostate cancer (33).

Programmed death-ligand 1 (PD-L1) and 2 (PD-L2), expressed by tumor cells and tumor-associated APCs (in tumor inflammatory microenvironment), are Programmed Death 1 (PD1) ligands and represent important immune checkpoint molecules. The interaction between the ligand and its receptor inhibits T-cell effector activity (34), primary T-cell response (35) and inducible Tregs suppression function (36). Given the critical role of PD1 in mediating T-cell exhaustion, anti-PD1 blocking antibodies have been developed to restore effector function of anti-tumor T cells. Monoclonal anti-PD1 antibodies, either alone or in combination with other agents, are used to manage advanced cancer stages such as melanoma, advanced squamous-cell lung carcinoma, NSCLC, advanced renal cell carcinoma, recurrent squamous cell carcinoma of the head and neck, advanced hepatocellular carcinoma and Hodgkin Lymphoma. Monoclonal antibodies against PD-L1 are used in NSCLC, advanced urothelial carcinoma, metastatic Merkel cell carcinoma (31).

### TCR Repertoire Diversity and Patients’ Response to CBI

The success of CBI depends on the presence of T cells able to recognize specific tumor antigens. The capacity of an individual to elicit an effective immune response is also directly correlated with tumor mutation load, which increases the likelihood of generating immunogenic neo-antigens and the chance to stimulate an anti-tumor immune response (37, 38). Thus, a broader TCR repertoire in patients receiving CBI would increase the chance of tumor antigen recognition and favorable long-term clinical outcome. Profiling TCR repertoire in patients before and after CBI has been used to assess dynamics of T-cell expansion and changes in T-cell clonotype diversity to predict and monitor patient response to therapy (39). Here we will highlight studies in which TCR diversity has been evaluated in the most commonly used CBI approaches: anti-CTLA4 e anti-PD1/PD-L1.

Anti-CTLA4 therapy shapes T-cell pool involved in anti-tumor recognition by indiscriminately broadening blood TCR repertoire (which also increase treatment side effects) (40, 41) and by increasing the number of tumor reactive T-cell clones (42, 43). Indeed, it has been shown that anti-CTLA4 therapy drove polyclonal expansion of TCR clones in tumor microenvironment (44) even those not specific for tumor antigens (45, 46). Analysis of pre-treatment TCR clonality in metastatic melanoma patients suggested that T-cell clonality within the tumor did not predict response to CTLA4 blockade (47). On the other hand, melanoma patients receiving anti-PD1 therapy showed increased TCR clonality (which was ten times greater in responders than in non-responders) and reduction in TCR diversity of intra-tumoral infiltrating lymphocytes (47).

Studies have found that higher peripheral blood TCR diversity is associated with improved clinical outcome in melanoma patients receiving anti-CTLA4 (48, 49) or anti-PD1 therapy (49). A similar study in melanoma patients observed that high pre-therapy clonality was associated with poor response to CTLA4, whereas it predicted good response to PD1 blockade (50). Higher baseline TCR diversity has been found to correlate with better disease control in patients with gastrointestinal cancers (51) and relapsed/refractory classical Hodgkin Lymphoma (52) receiving anti-PD1 therapy. Similarly, low T-cell clonality prior to anti-PD-L1 therapy and its increase in the periphery after immunotherapy has been associated with clinical benefits in patients with metastatic urothelial cancer (53). More recently, in a small group of patients affected by renal cell carcinoma receiving anti-PD1 therapy it was found which pre-treatment TCR diversity could not predict patients’ outcome and that restriction of TCR diversity early post-treatment (with following increase in TCR clonality) correlated with good response to therapy (54).

Thus, higher blood TCR diversity at baseline and increased TCR clonality following CBI have been associated with better clinical outcomes and increased survival in several studies, although not in all. Several factors can explain this discrepancy including the type of disease, the intra-tumoral mutation burden rate, patient previous therapies and method used to evaluate TCR diversity. In particular, the sample used to estimate TCR diversity could play a major role in the results. In addition to analysis in peripheral blood (which is representative of non-tumor and tumor specific TCRs), specific blood T-cell subsets could better help to characterize the association between TCR diversity and response to CBI. For instance, peripheral PD1+ T cells, in the case of anti-PD1 blockade therapy, which should be representative of tumor-specific T cells (46, 55), may represent an ideal target to assess TCR diversity. Indeed, contrary to analysis performed on bulk CD8+ T cells, melanoma patients with higher pre-treatment TCR diversity and reduced diversity post anti-PD1 treatment in CD8+ PD1+ showed longer progression free survival (46). In addition, higher pre-treatment TCR diversity on sorted PD1+ CD8+ T cells was also reported in those NSCLC patients with longer progression-free survival and better overall survival before anti-PD1/PDL1 therapy (56).

Overall, a broader T-cell receptor before CBI immunotherapy has been largely associated with a better clinical outcome in cancer patients (**Figure 1**). This may suggest that approaches that improve TCR repertoire diversity could render more patients receptive to CBI treatment.

As already discussed above, the decline of thymic function and the reduction of T cell repertoire diversity with age lead to holes in the repertoire that could compromise the efficacy of CBI. An increasing number of studies have been evaluating the possible association between age and response to CBI therapy in clinical studies, as well as in pre-clinical mouse models. In triple-negative breast cancer mouse model, one study demonstrated that young (8-12 weeks of age) and aged (>12 months of age) animals equally respond to anti-PD1. However, response to anti-CTLA4 therapy was significantly impaired in aged animals when tumor growth and survival were compared to young animals (57). Mechanistically, a lower number of infiltrating lymphocytes and a reduction in the expression of genes associated with antigen presentation and inflammation was observed in the tumor microenvironment of aged animals. Another study found that advanced age was associated with decreased overall survival in aged mice (>22 months of age) treated with anti-PD1 (58). Interestingly, the same study also demonstrated that survival of glioblastoma patients were inversely correlated with CBI therapy as such that older patients have worse survival compared to younger patients.

However, a large amount of clinical evidence suggests that CBI therapy remains effective even in patients over the age of 75 and similar clinical response has been observed when patients are stratified by age (59, 60). For instance, response to CBI immunotherapy has been found to be independent of age in patients affected with IV stage melanoma and treated with anti-CTLA4 (61). Surprisingly, other studies found that the response of melanoma patients to anti-PD1 was even better in older than in younger patients (62). A large meta-analysis of 19 CBI trials in advanced cancers found no significant association between age and response to therapy.

While multiple studies have found that age does not affect patients’ response to CBI, this possibility is still under debate. In particular, as several of the reported studies are limited due to the retrospective nature of the analysis, prospective clinical studies that would include larger cohorts of elderly patients would be required to answer this question.

## Manipulating Thymic Function to Enhance Efficacy of Cancer Immunotherapy

Although the thymus is extremely sensitive to injury, it maintains a remarkable capacity for repair (63, 64). Therapies aimed to enhance the regeneration of thymic function are an attractive strategy to restore a diverse T-cell pool and long-term immunity (65). Several studies explored the use of regenerative factors to enhance and broaden immune responses in individuals with thymic insufficiency and immunodeficiency resulting from infections, cancer therapies and immunosenescence (**Table 1** and **Figure 1**). Several therapies have been developed over time in preclinical models, some of which have been translated into clinical trials (5).

**Table 1:** Approaches, discussed in the review, to promote thymic function, their targets and their evaluation in clinical trials.

Therapeutic Approach	Targets cells	Clinical Translation		References
		Trial	Setting	
**Cytokines**				
IL-7	HSPCs, thymocytes, mature T cells	NCT01190111 NCT01241643 NCT00839436 NCT00684008 NCT00477321 **NCT04332653**	HIV HIV ICL HCT HIV **Advanced solid tumors (anti-PD1)**	(66) (67) (68) (69) (70) (70) (71) (72, 73)
IL-12	Thymocytes	Pre-clinical		(74) (75)
IL-21	HSPCs, thymocytes	Pre-clinical		(76) (77) (78)
IL-22	TECs	Pre-clinical		(79) (80)
RANKL	TECs	Pre-clinical		(81) (82) (83)
**Growth Factors**				
KGF	TECs	NCT00593554 NCT02356159 NCT03042585 NCT01233921 NCT01712945	HCT HCT HCT HCT MS	(84) (85) (86) (87) (88) (69) (89)
IGF-1	TECs	Pre-clinical		(90)
BMP4	TECs	Pre-clinical		(91)
**Hormones**				
Thymosin-α1	Thymocytes	NCT00580450 NCT00911443*	HCT Melanoma*	(92, 93) (94) (92) (95)
GH	TECs, thymocytes	NCT00287677 NCT00071240 NCT00050921 NCT00119769 NCT04375657	HIV HIV HIV HIV Immunosenescence	(96) (97) (98) (99) (100) (101)
Sex steroid ablation	TECs, HSPCs, thymocytes	NCT01746849 NCT01338987 **NCT03650894**	HCT HCT **Breast cancer (anti-PD1+ anti-CTLA4)**	(102) (63) (103) (104) (105) (106) (105) (107) (108, 109)
**Artificial Tissue**				
Artificial Thymus	TECs, thymocytes	Pre-clinical		(110) (111) (112, 113) (114)

In bold, clinical studies on CBI in combination with immune boosting strategy. (GH, growth hormone; HSPCs, hematopoietic stem and progenitor cells; KGF, keratinocyte growth factor; IL, interleukin; RANKL, receptor activator of nuclear factor-κB ligand; TECs, thymic epithelial cells; IGF1, insuline-like growth factor 1; HCT, hematopoietic cell transplantation; MS, multiple sclerosis; ICL, Idiopathic CD4+ lymphocytopenia; HIV, Human Immunodeficiency Virus).

*Clinical trial on the efficacy of thymosin-α1 in combination with dacarbazine in melanoma patients. Patients were subsequently treated with anti-CTLA4 in a separate study.

### IL-7

One of the most widely studied molecules with immune regenerative capacity is the cytokine IL-7, a key lymphopoietic factor with the ability to enhance the proliferation of lymphocytes and lymphoid precursors (66). Several pre-clinical studies demonstrated that IL-7 cytokine controls the size of the peripheral T-cell pool and plays an important role in regulating overall T-cell homeostasis (69, 72). Moreover, in patients enrolled in a phase I dose-escalation trial, recombinant human IL-7 (rhIL-7) administration safely induced polyclonal T-cell expansion, resulting in increased T-cell counts. Specifically, 4 of the 6 enrolled subjects showed a statistically significant increase in TCR repertoire diversity 1 week after the end of rhIL-7 treatment compared to their baseline levels in CD4^+^ and CD8^+^ populations (73). RhIL-7 therapy also augmented immune responses to weak antigens and spare Tregs expansion (73). In a phase I clinical trial (NCT00684008) in which the immune-regenerative properties of rhIL-7 were assessed in patients receiving T-cell-depleted allogeneic HCT, the majority of participants displayed enhanced TCR repertoire diversity that persisted several weeks after the end of rhIL-7 therapy (70).

A recombinant form of the human interleukin-7 (NT-I7), in combination with PD-L1 inhibition, will be assessed in a Phase 2 study for the treatment of NSCLC patients.

### KGF

Normal thymic T-cell development is strongly contingent on the regular maintenance of the stromal microenvironment. Thus, molecules that can promote recovery of stromal function, in particular of TECs, would support T-cell development and enhance T-cell reconstitution after damage. Keratinocyte Growth Factor (KGF) is a potent growth factor expressed by thymic stroma that binds to its receptor on TECs and induces thymic epithelial cells (TEC) proliferation (115). Given its peculiarity to protect thymic stromal compartment from damage, KGF administration has been exploited in thymic regeneration therapies (116). The impact of exogenous administration of KGF on TEC function and thymic regrowth has been extensively assessed in several mouse studies. It has been found that KGF administration significantly increased thymic cellularity in mouse models of aging and following acute damage caused by radiation or chemotherapy (88, 117). Moreover, several studies in mice and non-human primates demonstrated the efficacy of KGF for improving thymic-dependent T-cell recovery following HCT. In particular, KGF-treated animals showed increased numbers of T-cell receptor excision circles (TRECs), which measure thymic function in peripheral blood, up to 3 months following treatment (118).

### RANKL

The role of RANKL in the regeneration of the thymic microenvironment has been well characterized (82). Following thymic damage, RANKL induces up-regulation of lymphotoxin-α (LTα) which can bind to LTβ receptor on thymic epithelial progenitor cells and TECs, and promote their regeneration (83). Exogenous administration of recombinant RANKL boosts regeneration of TECs and improves T-cell progenitor homing and *de novo* thymopoiesis. Overall, these effects lead to enhanced T-cell development (81).

### Thymosin-α1

Thymic stroma, particularly TECs, also produces Thymosin-α1 (Tα1) that is able to increase thymocytes differentiation, boost T-cell function and promote immune recovery following hematologic insults (119). Several evidence in pre-clinical models have highlighted the immunomodulatory properties of Tα1; thus, this therapy has been studied in the clinic for the treatment of patients experiencing viral infections, immunodeficiency and hematological malignancies (92, 94). Treatment with Tα1 resulted in earlier appearance of pathogen-specific T-cell responses against pathogens such as cytomegalovirus and *Aspergillus* species after HCT (93). Interestingly, recent clinical studies also suggested that Tα1 may also have synergistic effects when used in combination with CBI. It has been shown that sequentially treatment with Tα1 and anti-CTLA4 significantly increased overall survival of melanoma patients (95).

### Growth Hormone and Insulin-Like Growth Factor-1

Growth Hormone (GH) is a small peptide hormone implicated in the regulation of hematopoietic function. It has been demonstrated that *in vivo* administration of a recombinant form of GH or insulin-like growth factor-1 (IGF1) (which represents one of the principal mediators of GH effects) can reverse thymic involution, increases TCR diversity and enhances recovery of hematopoietic compartments in patients with adult GH deficiency (90, 101, 120). Moreover, the administration of human recombinant GH in HIV-infected patients promoted thymic function and peripheral immune function (96, 99). A recent study also suggested that GH treatment can regenerate thymic tissue in healthy adults between 51 and 65 years of age (100). This treatment resulted in significant increase of both CD4^+^ and CD8^+^ naive T cells, and in decrease of PD1^+^CD8^+^ T cells (100).

### Ablation of Sex Steroids

Sexual dimorphism in the immune system is well recognized and it is broadly summarized with the concept that women tend to develop more autoimmune diseases than men, while men are more vulnerable to some infectious diseases. Sex hormones, and in particular androgens, heavily influence thymic function primarily through the regulation of TEC differentiation and function (121, 122). Studies in murine models demonstrated that age-related thymic dysfunction is faster in males than in females. Similarly, in humans, the rate of thymic involution is greater in males as demonstrated by evaluation of TRECs in patient peripheral blood (123, 124). As direct evidence of the close connection between sex hormones and thymic function, many pre-clinical studies have demonstrated that sex steroid ablation (SSA), by surgical or chemical approaches, transiently reverses thymic involution and promotes rejuvenation of lymphoid tissues. SSA induces thymic reconstitution and peripheral immune cells recovery after radiation, chemotherapy and HCT (63, 104–107). Recent studies have also shown that the effects of SSA are not restricted to the lymphoid lineage, as extensive regenerative signals are also directed towards the hematopoietic stem and progenitor cells and their niche (103, 108). While the underlying mechanisms are still not completely understood, experimental evidence demonstrated that some of the regenerative effects are mediated by the removal of the inhibitory effects of sex steroids, primarily of androgens, on endogenous B and T lymphopoiesis. The increase in androgens during life could also explain and contribute to the faster rate of thymic-involution observed after puberty. Most of our mechanistic understanding of the effects of hormones on thymic function is largely restricted to the effects of androgens in male subjects. However, recent studies have started characterizing genders differences in thymic function and in response to SSA (122, 125). It has been shown that, in female mice, age induces a higher degree of central tolerance imbalance characterized by the reduction of medullary TECs expressing the autoimmune regulator gene (AIRE), which could contribute to the increased risk of autoimmune disease observed in middle-aged women (126). In addition, middle-aged females are less affected by the regenerative effects triggered by SSA therapy compared to males but are more responsive when thymic regeneration was evaluated in response to acute thymic damage (125).

Interestingly, when transferred into the clinic, SSA has been shown to enhance neutrophil and lymphocyte recovery, thymic function and T-cell repertoire regeneration in patients receiving autologous and allogeneic HCT, independently from gender (109). Thus, while the precise mechanisms of action of SSA on lymphoid regeneration is still not completely understood, this approach represents an appealing therapy to enhance immune recovery in patients. Importantly, a clinical trial has been recently opened to evaluate if the regeneration of thymus and peripheral T-cell pool induced by SSA can enhance response to dual ICB with anti-PD1 and anti-CTLA4 therapy in metastatic breast cancer patients (127).

There is an incredible effort in the field to identify novel pathways and targets that can enhance thymic and immune recovery as the currently identified approaches are limited. In addition to IL-7, KGF, RANKL, SSA, GH and Thymosin α1, studies have found that other cytokines and growth factors have the potential to restore thymic function following immune insults. Administration of IL-12 induces thymocyte proliferation through increased IL-7 and IL-2 signaling (74). IL-21 delivery can also imprint regenerative signals to the thymus after immunological injuries such as glucocorticoid-induced thymic atrophy, aging and allogeneic HCT (76–78). IL-22 cytokine can mediate thymic regeneration by promoting TECs survival and proliferation through activation of STAT3 and STAT5 and expression of the antiapoptotic molecule *Mcl1* (80, 128). Furthermore, BMP4 produced by thymic endothelial cells can drive thymic regeneration by binding to its receptor expressed on TECs and stimulating the upregulation of *FoxN1* and its target genes (91). Critically, in patients with extensive thymic aplasia due to repetitive cycles of chemo or radiotherapy and/or aging, the presence of residual thymic tissue that could receive the regenerative signals and start organ recovery can be insufficient. In those conditions, the implant of artificial thymic tissue could represent an attractive alternative to repopulate the *naive* T-cell pool (110–114).

While some of the above-mentioned strategies have made some steps into the clinic, at present, there is no standard of care approach to promote immune reconstitution. In addition, their beneficial use in elderly cancer patients, which would greatly benefit from immune rejuvenating approaches, still requires additional research. Recent work observed that the increased disorganization and fibrosis of lymph nodes with age can limit the efficacy of thymic rejuvenation strategies (129). Thus, further studies are needed to determine whether secondary lymphoid organs are also rejuvenated with immune regenerative treatments, or whether approaches that could target both the thymus and the lymph nodes would represent a more effective therapy for immune recovery.

### Breaking Central Tolerance to Enhance CBI Efficacy

Central tolerance takes place in the thymus, where T cell clones that are reactive to self are deleted to protect against the development of autoimmunity. Although on the one hand, this process allows the elimination of T cells reactive against tissue-specific self-antigens (130), on the other hand, the majority of tumor cells, which express self-antigens, could be recognized by the same self-reactive T cells deleted by negative selection in the thymus (131). AIRE plays a crucial role in establishing central T cell tolerance controlling the expression of tissue-specific self-antigens in medullary TECs. AIRE deficiency leads to multiple autoimmune disorders in mice and patients. AIRE knock-out mice, which show expanded auto reactive T cell repertoire, have enhanced ability to mount anti-tumor response when challenged with syngeneic melanoma cells (132). Interestingly, a polymorphism in AIRE, which can decrease the stability of the mRNA, has been associated with protection from melanoma (133). Thus, while protecting against autoimmunity, AIRE also limits antitumor immunity. Thus, recent studies have been investigating alternative approaches to enhance T cell-mediated antitumor immunity and response to CBI, which are based on temporary disruption of central T cell tolerance through the inhibition of AIRE (131). Evidence of this approach has been provided in pre-clinical settings by the infusion of anti-RANKL antibody, which depleted AIRE-expressing TEC in the thymus and allowed self/melanoma-reactive T cells to escape negative selection and increase in the peripheral pool. Combination of anti-RANKL and anti-CTLA4 antibody therapy enhanced anti-tumor response and survival after melanoma challenge (134). Similarly, the use of anti-RANKL/PD-1 dual targeting antibody has been shown to promote anti-tumor response in pre-clinical tumor models (135).

While the depletion of AIRE+ TECs and the suppression of central tolerance after anti-RANKL therapy could play an important role in the enhanced anti-tumor activity when combined with CBI, further studies are needed to better characterize the contribution of RANKL antagonism on the tumor microenvironment.

## Conclusions

T-cell immunity is critical to control cancer occurrence and relapse. A more diverse TCR repertoire increases the likelihood of tumor-antigen recognition and of mounting an effective immune response. As the thymus represents the primary site of T-cell development and its function directly shapes the peripheral TCR diversity, robust residual thymic function in adult life can be associated with greater chance of establishing effective tumor immunity. However, direct evidence of the connection between thymic function and cancer is still under investigation. While thymic boosting approaches can have an immediate impact to enhance immune reconstitution after cytoreductive therapies, which would significantly reduce morbidly and improve survival in HCT patients, their potential use to extend the benefit of CBI is just beginning to be investigated. Indeed, although CBI has tremendously improved the chances of survival of cancer patients, a large proportion of them do not respond. Would patients with greater residual thymic functionality have greater chance to respond to CBI? In addition, multiple studies have investigated the use of TCR-seq as a predictive and prognostic tool for patient’s response to CBI. Broader TCR diversity has been linked to greater response to CBI in multiple studies. However, the methodology is associated with significant cost and methodological bias. Thus, can the assessment of thymic function, for example through the evaluation of TRECs or recent thymic emigrants, better and more precisely stratify patients that could benefit from CBI? Clinical trials in progress will be fundamental to answer to these questions and explore these intriguing possibilities.

## Author Contributions

AC and CD conducted the literature review, created the figures, and wrote the bulk of the manuscript. FL contributed to writing and editing. EV conceived the review and contributed to the planning, editing and writing. All authors contributed to the article and approved the submitted version.

## Funding

EV was supported by grants from the Amy Strelzer Manasevit Research Program; the Italian Association for Cancer Research (AIRC); and the Italian Ministry of Health (“Ricerca Corrente”). FL was supported by grants from AIRC (Special Program Metastatic disease: the key unmet need in oncology 5 per mille 2018 Project Code 21147 and Accelerator Award 2017 INCAR); Ministero dell’Istruzione, dell’Università e della Ricerca, PRIN ID 2017 WC8499_004; Ministero della Salute, RF-2016-02364388.

## Conflict of Interest

The authors declare that the research was conducted in the absence of any commercial or financial relationships that could be construed as a potential conflict of interest.

## Publisher’s Note

All claims expressed in this article are solely those of the authors and do not necessarily represent those of their affiliated organizations, or those of the publisher, the editors and the reviewers. Any product that may be evaluated in this article, or claim that may be made by its manufacturer, is not guaranteed or endorsed by the publisher.
